# Agreement Between Standard Optical Coherence Tomography and Optical Coherence Tomography-Based Angiography in Estimating Retinal Nerve Fiber Layer Thickness

**DOI:** 10.4274/tjo.galenos.2020.18488

**Published:** 2020-10-30

**Authors:** Hayati Yılmaz, Mehmet Talay Köylü, Alper Can Yılmaz, Ali Hakan Durukan, Yusuf Uysal

**Affiliations:** 1 University of Health Sciences Turkey, Ümraniye Training and Research Hospital, Clinic of Ophthalmology, İstanbul, Turkey; 2 University of Health Sciences Turkey, Gülhane Medical Faculty, Department of Ophthalmology, Ankara, Turkey

**Keywords:** Glaucoma, optical, coherence, angiography, agreement

## Abstract

**Objectives:**

To investigate the agreement between optical coherence tomography (OCT) and OCT-based angiography (OCT-A) in estimating retinal nerve fiber layer thickness (RNFLT) and evaluate the associations between peripapillary vessel density (VD) and RNFLT measurements obtained with both devices.

**Materials and Methods:**

The AngioVue (Optovue Inc., Fremont, CA, USA) and Spectralis (Heidelberg Engineering, Heidelberg, Germany) images of 325 patients were screened retrospectively. RNFLT values were recorded using both devices. The intraclass correlation coefficient (ICC) and Bland-Altman plots were obtained to investigate the agreement between the devices. Age- and intraocular pressure-corrected associations between VD and RNFLT measured by the two devices were analyzed using linear regression models.

**Results:**

ICC revealed excellent agreement for global, superior, inferior, and temporal RNFLT and good agreement for the nasal quadrant (ICC=0.895, 0.936, 0.923, 0.887, and 0.614, respectively). The Bland-Altman plots showed poor agreement for all measurements with a large span of limits of agreement and significant proportional bias (p<0.05). VD was found to be strongly associated with the RNFLT measurements of both devices (p<0.001).

**Conclusion:**

The disagreement between the devices should be considered in clinical practice, and the data should not be used interchangeably. The association of the peripapillary VD with RNFLT using both devices indicated that RNFLT assessed by the AngioVue could be used in glaucoma management along with VD.

## Introduction

Optical coherence tomography (OCT), first invented in 1991, allows imaging and analysis of the retinal nerve fiber layer (RNFL).^1^ Given the fact that functional visual field loss cannot be detected until up to 40% RNFL loss, analysis of the RNFL has great importance for the early detection of glaucoma.^2,3,4,5,6^

With technological advances, time domain OCT, which has a scanning speed of 400 A-scans/s, has been replaced by spectral domain OCT (SD-OCT), with an improved scanning speed of 29,000 to 55,000 A-scans/s, in ophthalmological practice.^7^ Recently, the advent of OCT angiography (OCTA) has enabled the noninvasive visualization of the retinal and peripapillary vessels, as well as analysis of structural parameters such as RNFL thickness (RNFLT) in the same scan.^8,9^ Using OCTA, the reduction of vessel density (VD) and blood flow index in glaucomatous eyes has been well documented in numerous studies.^10,11^ Furthermore, it was established by previous studies that OCT and OCTA both have excellent repeatability and reproducibility.^12,13,14,15,16^

There are limited data about the structural measurements of peripapillary parameters such as RNFLT using the OCTA scanning module. It is very important for clinicians to determine whether OCTA could be used only for the vascular network assessment of the retina and optic nerve head (ONH) and whether this can replace traditional OCT for monitoring glaucoma patients. Therefore, the aim of the present study was to investigate the agreement between SD-OCT scans and SD-OCT-based OCTA system scans for RNFLT measurement in subjects with primary open-angle glaucoma (POAG).

## Materials and Methods

The present study was conducted after receiving the approval of the Institutional Non-interventional Research Ethics Committee of The Health Sciences University and was conducted in agreement with the principles of the Declaration of Helsinki. The study was designed retrospectively, and patients with a diagnosis of POAG or glaucoma suspect (GS) who visited our glaucoma clinics between January 2018 and August 2019 and underwent OCT and OCTA scans during this visit were identified from the hospital records. The exclusion criteria were as follows: age under 18 years, refractive error greater than ±3 diopters, presence of any other ophthalmological pathology that could confound the assessment results (e.g., diabetes or hypertensive retinal diseases, amblyopia, optic nerve anomalies, optic neuropathies other than glaucoma, and age-related macular degeneration), SD-OCT signal strength <20 for Spectralis, and OCTA signal strength <70 for AngioVue.

### Assessment of RNFLT Using SD-OCT

Spectralis OCT (version 4.0) (Heidelberg Engineering, Heidelberg, Germany) was used for the measurement of RNFLT. This device has an A-scan rate of 40,000/s using a light source of 820 nm. An en face image focusing on the ONH was generated using a confocal scanning laser ophthalmoscope, and after a 3.4-mm circle was centered on the ONH, 15 images were acquired under high resolution settings and averaged automatically by the built-in software. After the scan was completed, global and quadrantal RNFLT values were recorded in accordance with the temporal-superior-nasal-inferior-temporal (TSNIT) chart.

### Assessment of RNFLT Using OCTA

OCTA scan acquisition was performed using an AngioVue OCTA system (Optovue Inc., Fremont, CA, USA) with an A-scan rate of 70,000/s using a light source of 840 nm. En face images were acquired focusing on the ONH (4.5 mm x 4.5 mm) using the Angio Disc QuickVue module. Each scan contained 400x400 A-scans with two following B-scans at each fixed location. To reduce motion artifacts, each image consisted of one orthogonal horizontal and vertical scan.

OCTA provides RNFLT and peripapillary VD data in the same scan. After the scanning was completed, in addition to the VD of the global retinal peripapillary capillary plexus (RPCP), global and quadrantal RNFLT values were recorded in accordance with the TSNIT chart.

### Statistical Analyses

Quantitative variables were expressed as mean and standard deviation (SD), and qualitative variables as percentages. The paired t-test was used to compare the results of the two devices. The magnitude of the disagreement between the Spectralis and the AngioVue data was estimated as the mean absolute difference of the global and quadrantal RNFLT values. The agreement between the corresponding Spectralis and AngioVue data was calculated using intraclass correlation coefficient (ICC). Absolute ICCs based on the mixed model analysis of variance were used in the present study. An ICC value less than 0.4 was considered to indicate poor agreement, 0.4 to 0.75 as fair to good agreement, and a value greater than 0.75 as excellent agreement.^17^ ICC calculations were performed before and after the subjects were divided into the POAG and GS groups. The Shapiro-Wilk test was used to determine whether the differences in corresponding Spectralis and AngioVue data were normally distributed. Bland-Altman plots were used to assess the agreement between Spectralis and AngioVue in terms of RNFLT measurements. One-sample t-test was performed to reveal the differences before the Bland-Altman plots were created.^18^ Then, the linear regression analyses of the Bland-Altman plots were performed to determine the significance of the proportional biases. Age- and intraocular pressure (IOP)-corrected linear regression models were created to analyze the associations between RNFLT data and the small VD of the RPCP. The results of the regression results were obtained with coefficients (B), 95% confidence intervals (CI), and p values. All statistical analyses were undertaken using IBM SPSS Statistics version 21 (IBM Corp., Armonk, NY, USA). A p value less than 0.05 was considered statistically significant.

## Results

Of the 386 patients that met the inclusion criteria, a total of 325 patients (52% female, mean age 61.4±18.7 years) were included in the study, and only 1 eye of each patient (with better SD-OCT and OCTA quality) was included in the statistical analysis. Of these 325 eyes, 218 were diagnosed as having POAG and 107 as GS.

Table 1 presents the mean global and quadrantal RNFLT values obtained with AngioVue and Spectralis, the mean differences between the measurements of the two devices, and the results of the paired t-test. The devices significantly differed in all RNFLT parameters (p<0.001). AngioVue tended to measure RNFLT thicker than Spectralis in all quadrants. The global, superior, nasal, inferior, and temporal RNFLT measurements of the two devices were all positively and strongly correlated (R=0.948, 0.908, 0.764, 0.920, and 0.824, respectively).

The mean small VD of the RPCP was 47.97% for the whole sample, 45.84% for the POAG patients, and 51.24% for the GS patients. VD was significantly lower in the POAG group compared to the GS group (p<0.001, Student’s t-test).

### Agreement Between SD-OCT and OCTA

The results of the ICC analyses are presented in Table 2. There was excellent agreement between the two devices for all RNFLT measurements (ICC=0.895 for global RNFLT, 0.936 for superior, 0.923 for inferior, and 0.887 for temporal) except the nasal quadrant, for which the agreement was good (ICC=0.614). The rates of agreement were higher for the POAG patients than GS cases (Table 2).

**Figure 1:**
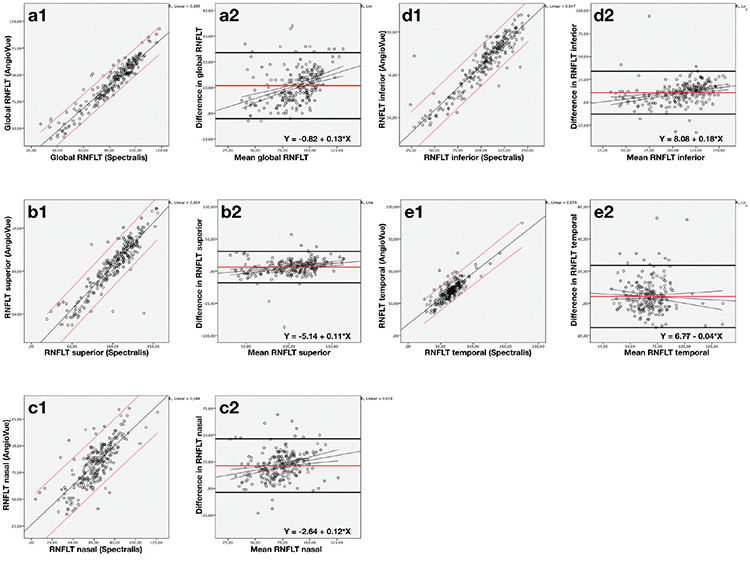
Scatter plots of AngioVue versus Spectralis OCT for RNFLT (column 1) and the Bland-Altman plots for the agreement between AngioVue and Spectralis for the RNFLT (column 2). Scatter plots were given with the regression line (black line) and the 95% limits of agreements (red lines). Bland-Altman plots were given with the mean of the difference (red bold line), 1.96 SDs (black bold lines), and the regression lines (black thin lines). RNFLT scatter and Bland-Altman plots for global (a1,a2), superior (b1,b2), nasal (c1,c2), inferior (d1,d2), temporal (e1,e2) RNFLT values OCT: Optical coherence tomography, RNFLT: Retinal nerve fiber layer thickness, SD: Standard deviation

Figure 1 presents the scatter plot of AngioVue versus Spectralis in terms of global, superior, nasal, inferior, and temporal RNFLT along with the Bland-Altman plots of these parameters. The Bland-Altman plots showed that the mean bias ± SD between the measurements of AngioVue and Spectralis was 10.74±6.56 for global RNFLT, and 6.35±12.47, 21.39±12.78, 10.53±11.91, and 4.29±9.86 for superior, nasal, inferior, and temporal RNFLT, respectively. The limits of agreement (LOA) were -2.11 -23.59 for global RNFLT, -18.09 -30.79 for superior, -3.67 -46.45 for nasal, -12.99 -33.69 for inferior and -15.03 -23.61 for temporal RNFLT. The Bland-Altman plots revealed the slope of the regression line as 0.13, 0.11, 0.18, 0.12, and -0.04 for global, superior, nasal, inferior, and temporal RNFLT, respectively, indicating that AngioVue tended to provide thicker RNFLT values than Spectralis except for the temporal quadrant, where the opposite result was obtained. The linear regression analyses of the proportional bias were all statistically significant except for the temporal quadrant (p<0.001 for global, inferior, and nasal RNFLT, p=0.014 for superior RNFLT and p=0.407 for temporal RNFLT). The wide spans of LOA and the statistically significant proportional biases suggested poor agreement between AngioVue and Spectralis. However, for the temporal quadrant, the disagreement between the two devices seemed to be much more tolerable.

### Associations with RPCP VD

The results of the regression models are given in Table 3. Global and all quadrantal RNFLT values obtained by both AngioVue and Spectralis were strongly and independently associated with the small VD of the RPCP. Global RNFLT had the closest relation with the small VD of the RPCP (B=0.327 for AngioVue and 0.382 for Spectralis).

## Discussion

The present study investigated the agreement between two different SD-OCT scanning systems, Spectralis OCT and AngioVue, in terms of RNFLT measurements. Although these devices use similar technologies to measure RNFLT and they are both systems based on SD-OCT, the differences in their built-in algorithms caused important differences, indicating that the data should not be used interchangeably. Age- and IOP-controlled associations of the peripapillary VD with RNFLT measurements assessed by these devices were also evaluated in this study. The RNFLT measurements of both devices were found to be strongly associated with the small VD of the RPCP.

Previous studies assessing the agreement between OCT devices similar to our study found better agreement for global thickness.^19,20,21^ The better agreement for global RNFLT compared to quadrant thicknesses may be related to the small centralization errors around the ONH. It has been shown that quadrantal measurements may result in greater errors in noncentered scans.^22^ Hong et al.^23^ compared RNFLT measurements assessed by swept-source OCT (SS-OCT, Triton, Topcon) with SD-OCT (3D-OCT-2000, Topcon). They found that the agreement between these devices was excellent; however, they suggested that the clinician should be aware of measurement errors, especially in patients with retinal diseases. Koh et al.^24^ investigated the agreement between SD-OCT (Cirrus HD-OCT, Carl Zeiss Meditec) and OCT/scanning laser ophthalmoscopy (OCT/SLO, OPKO/OTI) and found differences in RNFLT measurements; however, they also suggested that the devices were similar in terms of the detection of glaucomatous damage. Similarly, Mwanza et al.^25^ compared 3 different SD-OCT devices (Cirrus HD-OCT, Spectralis OCT and RTVue) and reported different RNFLT values but noted that the dynamic range and the number of steps to the RNFL floor were similar between the devices. Leite et al.^19^ also compared the same 3 devices and concluded that these devices could not be used interchangeably.

The only study which investigated the agreement in RNFLT measurements between the traditional OCT system (Cirrus HD-OCT, Carl Zeiss Meditec) and OCTA (Plex Elite 9000, Carl Zeiss Meditec) as in our study was that of Tan et al.^9^, who found excellent agreement in terms of the global, superior, and inferior quadrants and good agreement in the temporal and nasal quadrants. The authors concluded that RNFLT could be sufficiently extracted from wide-field OCTA scans. In our study, the agreement was also excellent for the global, superior, inferior, and temporal RNFLT and good for the nasal quadrant.

While investigating the agreement between instruments, the intra-subject repeatability of measurements should also be considered. Previous studies investigated the repeatability of measurements for numerous commercially available OCT devices and the intra-test variability was found to be approximately 5 µm.^21,26,27^ With an intra-test variability of 5 µm for each device, even if the instruments have perfect agreement, an error of approximately 10 µm (±5 µm) in the agreement could be expected.^19^ In the present study, the span of the LOA of the global RNFLT was approximately 25 µm, more than twice the expected error. The spans of the LOA of the quadrants were even wider than that of the global RNFLT, again indicating that the AngioVue and Spectralis data could not be used interchangeably.

There were fixed biases between the measurements in all corresponding parameters. The AngioVue measurements were consistently thicker than those of Spectralis. There were also proportional biases, indicating that the differences in the RNFLT measurements between the instruments varied according to the actual thickness of RNFL. AngioVue consistently measured RNFLT thicker than Spectralis in all quadrants, but this difference was even greater where the RNFL was thicker. AngioVue and Spectralis seemed to agree more in cases with a thinner RNFL. This was also supported by the much higher ICCs of the POAG patients compared to the GS subjects. Similar proportional biases were observed in previous studies.^19,23,24,28,29^ However, no assumption can be made regarding the accuracy of the measurements without a direct comparison to histologic measurements.

The differences between AngioVue and Spectralis were greatest in measurements of the nasal quadrant. There were strong correlations between the corresponding measurements of the two devices; however, the correlation of the nasal quadrant measurements was the weakest. In addition, ICCs revealed good agreement for the nasal quadrant but excellent agreement for all the remaining quadrants, which is consistent with the findings of previous studies.^9,19,20,30^ The reason for the greater difference in the nasal quadrant measurements might be the use of different incidence angle of the laser beam by different devices.^19^

Small VD in the RPCP was found to be associated with RNFLT in all quadrants for both AngioVue and Spectralis. She et al.^31^ showed that the peripapillary VD was closely related to RNFLT and concluded that OCTA could be valuable for detecting glaucomatous damage. In contrast to She et al.^31^, Holló^32,33^ conducted a 2-year follow-up study and reported that peripapillary VD did not support glaucomatous progression; however, after removing the large vessels, peripapillary VD could be helpful in detecting progression. The built-in software of AngioVue automatically removes the large vessels and calculates only small VD. The associations of the RNFLT measurements of both devices with RPCP VD were nearly the same. This indicates that RNFLT assessed with OCTA is adequate for use in the management of glaucoma.

## Conclusion

In conclusion, even with the excellent agreement revealed by ICC, the wide spans of LOA and the significant proportional biases suggest poor agreement between AngioVue and Spectralis, and therefore the data obtained from these instruments should not be used interchangeably. However, given the fact that VD in the peripapillary area is highly associated with glaucomatous damage and that the results of the present study revealed strong correlations between RNFLT and peripapillary VD for both AngioVue and Spectralis measurements, the RNFLT values of OCTA could be used for monitoring RNFLT in addition to VD in patients with glaucoma.

## Figures and Tables

**Table 1:**
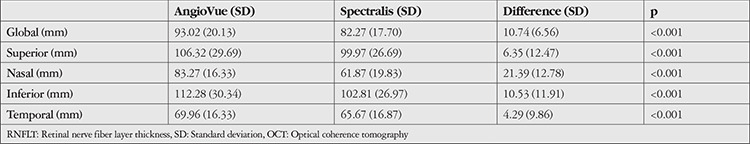
Mean values of the AngioVue and spectralis OCT measurements of the RNFLT and the paired t-test results

**Table 2:**
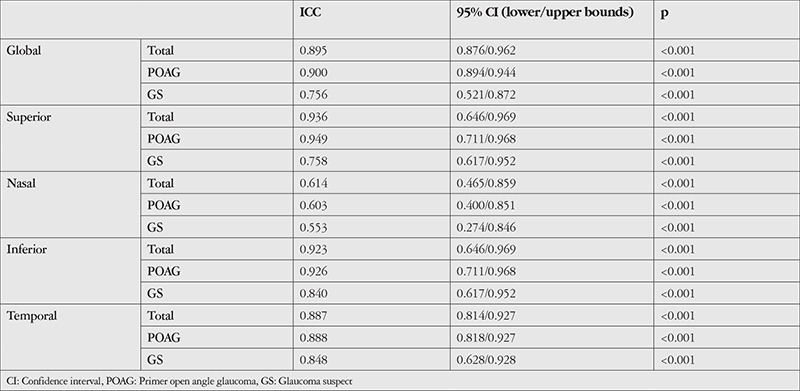
Intraclass correlation coefficient (ICC) values between AngioVue and spectralis measurements

**Table 3:**
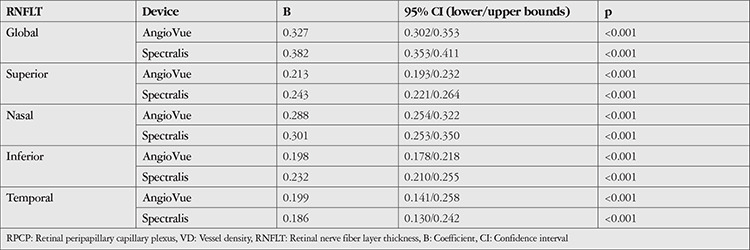
The linear regression models for the RPCP small VD
